# Exploring the impact of telemedicine in chronic patients from diverse socioeconomic contexts: systematic review of qualitative studies

**DOI:** 10.3389/fpubh.2024.1510735

**Published:** 2025-01-29

**Authors:** Yuzhe Li, Qifang Shi, Jing Yang, Li Ran

**Affiliations:** Faculty of Nursing, Health Science Center, Xi'an Jiaotong University, Xi'an, Shaanxi, China

**Keywords:** telemedicine, chronic disease management, electronic health, qualitative meta-synthesis, self-management

## Abstract

**Background:**

Telemedicine has a positive impact on improving health literacy and disease management ability of chronic disease patients and reducing the medical burden. However, telemedicine still has many problems in chronic disease management. We should actively solve the problems and maximize the functions of telemedicine.

**Objective:**

To explore the application and improvement of telemedicine in chronic disease self-management to provide important insights from functional module analysis for stakeholders, healthcare professionals, and policymakers to promote the development of telemedicine in chronic disease management.

**Design:**

We conducted a systematic review and qualitative synthesis of five English databases including PubMed, Embase, Web of Science, Cochrane Database of Systematic Reviews, and Scopus, as well as three Chinese databases: China National Knowledge Infrastructure (CNKI), WanFang and VIP database. Databases were searched from inception until November 12, 2024.

**Methods:**

This review is reported in accordance with guidelines for Enhancing Transparency in Reporting the Synthesis of Qualitative Research (ENTREQ). Two reviewers independently performed study selection, data extraction, and quality assessment using the Joanna Briggs Institute's key assessment tools for qualitative research. Thematic analysis was used for data synthesis.

**Results:**

A total of 35 studies were included, and the contents were refined and summarized into 8 new subthemes. Then, four themes were synthesized: Reminder and supervisor, Access to knowledge, Transition in medical treatment mode, and Emotional support platform.

**Conclusion:**

By improving information quality, developing diverse functions, and constructing multidisciplinary coordination mechanisms to meet the needs of patients with chronic diseases, improve the medical service system, maximize the function of telemedicine, and enhance the stickiness of patients to use telemedicine.

## 1 Introduction

Non-communicable diseases, also known as chronic diseases, are the leading cause of death worldwide. Chronic diseases kill 41 million people each year, which is equivalent to 74% of all deaths worldwide (1). Chronic diseases have a negative impact on patients, families, and society due to the demands of long-term treatment and management (2). With the progress and development of science and technology, digital technology has been widely used in medicine. Especially during the COVID-19 Pandemic, the use rate of telemedicine has increased significantly. Patients have remote consultations with doctors through telephone and video calls. This approach not only avoids the risk of cross-infection but also reduces the burden on the hospital (3). After the COVID-19 pandemic, telemedicine has gradually become a normal medical service mode, especially in the fields of chronic disease management and older patients care. Internet of Things (IoT) technology combined with wearable devices makes remote monitoring more convenient and accurate (4). Telemedicine not only allows patients to remotely monitor and consult patients by using wearable devices or mobile phones but also enables doctors to efficiently and intelligently complete disease diagnosis treatment and teaching by using electronic medical records and artificial intelligence (AI) (5). At present, many studies have confirmed that telemedicine can improve the management and monitoring of patients with chronic diseases (6), increase the accessibility and efficiency of medical services, reduce the incidence of chronic diseases, and effectively improve health literacy and disease management ability of patients with chronic diseases and improve their quality of life (7).

Despite the great potential of telemedicine in chronic disease management, there are still many problems. The study found that the low digital literacy of patients and the difficulty of technical operations hindered the development of telemedicine. At the same time, telemedicine also poses challenges for healthcare professionals and policymakers (8). Telemedicine increases healthcare professional's workload and puts higher demands on their service level and the allocation of national medical resources. However, most of the previous studies focused on the experience and preferences of patients, and there was a lack of systematic research on the application function of telemedicine (9, 10). Cultural background significantly impacts whether patients are willing to use telemedicine services. In areas with a strong traditional culture, patients may prefer face-to-face visits, believing that in-person contact with a doctor is the most reliable form of treatment. The lack of face-to-face interaction in telemedicine can lead to trust issues, especially for older people and low-income groups (11). Efficient and stable network connection is an inevitable requirement of telemedicine, and the lack of mobile devices will hinder the popularization of telemedicine. Compared with developing countries, developed countries have obvious technological advantages, which is conducive to the implementation of telemedicine (12). The study aims to explore the application and improvement of telemedicine in chronic disease self-management to provide important insights from functional module analysis for stakeholders, healthcare professionals, and policymakers to promote the development of telemedicine in chronic disease management.

## 2 Methods

### 2.1 Study design and search strategy

A systematic review and thematic synthesis of qualitative research was carried out under the guidance of the framework and reporting guidelines for Enhancing transparency in reporting the synthesis of qualitative research (ENTREQ) (13) (Supplementary material). Searches included five English databases in PubMed, Embase, Web of Science, Cochrane Database of Systematic Reviews, and Scopus, as well as three Chinese databases: China National Knowledge Infrastructure (CNKI), WanFang, and VIP database. Databases were searched from inception until November 12, 2024. The search strategy included a comprehensive keyword combination of chronic disease, telemedicine, and qualitative research. To prevent omissions, we also screened references for included studies and relevant systematic reviews. The search strategy is given in Supplementary material.

### 2.2 Inclusion and exclusion criteria

Inclusion criteria: (1) Study participants: patients with at least 1 common chronic disease; (2) Context of the study: patients with home-based disease management or interviewed after participating in the telehealth experiment during follow-up. (3) Phenomenon of interest: experience, functional application, and suggestions for improvement of telemedicine; (4) Study methods: qualitative research, including descriptive research, phenomenology, ethnography, grounded theory, narrative or thematic analysis, and so on.

Exclusion criteria: (1) Repeated publication, incomplete data information; (2) Non-Chinese and English literature; (3) Conference or review articles; and (4) Full text is not available.

### 2.3 Study selection and data extraction

It was independently conducted by 2 researchers in the search team who were trained in evidence-based practice methods. The researchers screened the retrieved study titles and abstracts from the literature search results to exclude studies that did not meet the inclusion criteria. The researchers then further screened the included studies by reading the full text and sought comprehensive information from the authors of the included articles when necessary. If two researchers disagreed, a third researcher was consulted. Use a pre-designed table to extract key descriptive characteristics of the included study, including author, year of publication, country, study purpose, participant characteristics, method of data analysis, and key findings. The authors would be contacted via email for additional or missing information if required. The extracted text was entered verbatim into NVivo 12 plus for management and analysis.

### 2.4 Quality assessment

The Joanna Briggs Institute Qualitative Assessment and Review tool was used to assess the quality of included studies. The scale consists of 10 questions answered with “yes,” “no,” and “unclear.” These 10 questions are designed to help the researcher conduct a quick and systematic evaluation of the essay. If all the answers are “yes,” the criteria are fully met and the study is rated as A; if the evaluation criteria are partially met, it is rated as B; all answers are “no,” meaning they do not meet the criteria at all and are graded C. Studies rated C were excluded. Two researchers independently assessed the quality of each study, and a third researcher was contacted for judgment if there was a disagreement.

### 2.5 Data synthesis

Qualitative data were analyzed using Thomas and Harden's three-stage thematic synthesis approach (14). The included studies and extracted data were read repeatedly by three researchers to gain a full understanding before synthesis. In the first stage, all the experiences and preferences associated with the use of m-health for patients with chronic diseases were summarized and coded. In the second stage, the codes are grouped by comparing the similarities and differences of the data in the first stage, grasping the connections, merging similar themes, and creating new descriptive themes. In the final stage, the descriptive topics from the previous stage are re-examined and similar topics are grouped into a comprehensive result (analytical topics). In this process, sub-themes and main themes, namely descriptive themes and analytical themes, are generated, and new interpretations of phenomena are obtained. The first stage of the coding process was carried out by two researchers. Descriptive and analytical themes are identified by one researcher. Subsequently, the data analysis process was checked by the entire research team to ensure consistency in the interpretation of the results and adequacy of the topics analyzed.

## 3 Results

### 3.1 Description of studies

**Figure 1:**
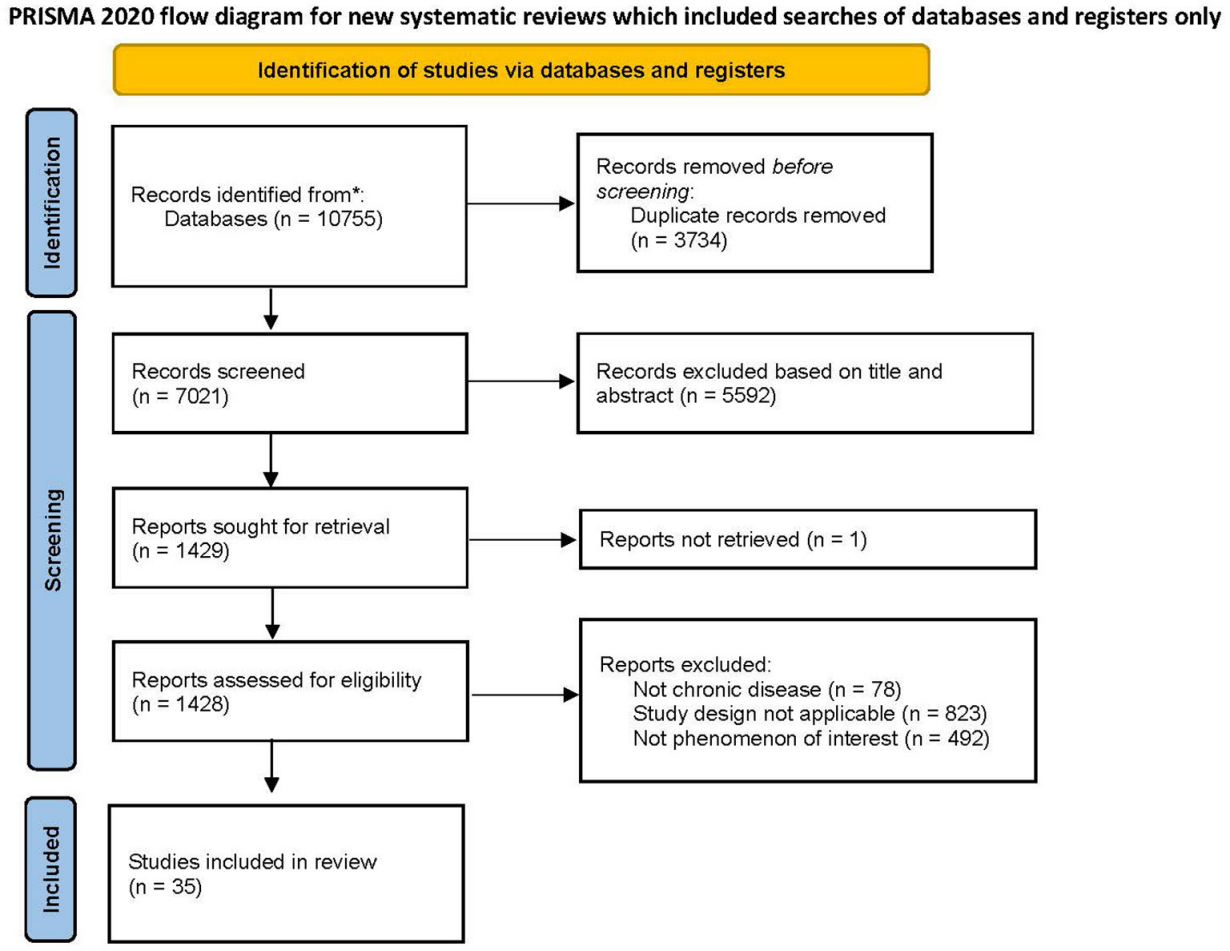
PRISMA diagram presenting process of selection and study screening.

The initial search identified 10,755 articles, and 7,021 articles were left after eliminating the duplicates. Finally, 35 articles were included in this meta-synthesis through the screening of inclusion criteria. The included studies were published between 2008 and 2024 and included a total of 768 patients with chronic disease and 173 healthcare professionals. The search results and process are shown in Figure 1.

### 3.2 Description and quality assessment of included literature

The majority of studies were from high-income countries, including the United Kingdom (10/35, 29%) (15–24), China (9/35, 26%) (25–33), United States (4/35, 11%) (34–37), Sweden (3/35, 9%) (38–40), Canada (3/35, 9%) (41–43), and each from the Netherlands (44), Australia (45), Denmark (46), Belgium (47), Germany (48), and Norway (49) (1/35, 3%). mHealth technologies were applied to assist the management of the following CNCDs: diabetes (4/35, 11%) (21, 31, 34, 47), chronic obstructive pulmonary disease (10/35, 29%) (16, 17, 19, 23, 24, 28, 29, 32, 46, 49), cardiovascular disease (9/35, 26%) (18, 20, 22, 25–27, 39, 40, 42), kidney disease (1/35, 3%) (35), and multiple chronic conditions (11/35, 31%) (15, 30, 33, 36–38, 41, 43–45, 48). The analytic approaches followed in these qualitative studies included thematic analysis (7/35, 20%) (20, 21, 32, 34, 35, 41, 46), content analysis (11/35, 31%) (31, 33, 37–39, 42–44, 47–49), constant comparative method (2/35, 6%) (22, 24), framework analysis methods (FAMs) (5/35, 14%) (15–18, 36), interpretative phenomenological analysis (2/35, 6%) (19, 40), Colaizzi's seven-step analysis (6/35, 17%) (25–30), and non-specific method (2/35, 6%) (23, 45) (Table 1).

**Table 1:** Characteristics of the included studies.

**References**	**Country**	**Aim of study**	**Participants**	**Sample**	**Study design**	**Main results**
Lu et al. (33)	China, Taiwan	To describe use of home telehealth care as an alternative for chronic disease management from users' s perspective	Patients with hypertension, diabetes, or both	*N =* 20 Age range: 50–86 Mean age: 67	Qualitative content analysis (Graneheim & Lundman)/Focus group discussion/Face-to-face interviews	• Perceived support and security • Enhanced disease self-management • Concerned with using the devices • Worries about the cost by patients
Chang et al. (31)	China, Taiwan	To investigate the older diabetes patients' acceptance and perceived benefits of telehealth service	Patients with diabetes	*N =* 18 M = 6 F = 12 Age range: 65–91 Mean age: 77.6	Qualitative content analysis/One-on-one semi-structured interviews	• Initial trial encouragement from the doctors, nurses, and financial incentives • Enhanced self-management capability through continuous device use for better outcomes • Ambivalent feelings regarding dependence on others for problem-solving • Consideration for continual technology use for an uncertain future
Fairbrother et al. (17)	UK	To explore patient and professional views on self-management in the context of telemonitoring in chronic obstructive pulmonary disease (COPD)	Patients with COPD and healthcare professionals	*N =* 70 (Patients = 38 Age range: 44–85 Mean age: 67.5, HCPs = 32)	Semi-structured interviews/Framework approach (Ritchie & Lewis)	• Knowledge and empowerment • Accessibility and reassurance • Medicalization, compliance and dependence • The parameters of empowerment
Fairbrother et al. (16)	UK	To explore the views of patients and professionals on telemonitoring and investigate the perceived impact of telemonitoring on continuity of care	Patients with COPD and healthcare professionals	*N =* 70 (Patients = 38 Age range: 44–85 Mean age: 67.5, HCPs = 32)	Semi-structured interviews/Framework approach (Ritchie & Lewis)	• Reassurance, accessibility and trust • Knowing the patient • Discontinuities • Issues of cost and continuity
Fairbrother et al. (18)	UK	To understand the views of patients and professionals on the acceptability and perceived usefulness of telemonitoring in the management of chronic heart failure in the context of day-to-day care provision	Patients with chronic heart failure	*N =* 18 M = 11 F = 7 Age range: 50–80 Mean age: 74	Semi-structured interviews/Framework approach (Ritchie & Lewis)	• Information, support and reassurance • Compliance and dependence • Changes and challenges • Determining the criteria for patient applicability to telemonitoring • Continuity of care
Hanley et al. (20)	UK	To explore the experiences of patients and professionals taking part in a randomized controlled trial (RCT) of blood glucose, blood pressure (BP) and weight telemonitoring in type 2 diabetes supported by primary care, and identify factors facilitating or hindering the effectiveness of the intervention and those likely to influence its potential translation to routine practice	Patients with type 2 diabetes	*N =* 33 (Patients = 23, Nurses = 6, Doctors = 4)	Interpretive descriptive approach/Semi-structured interviews/Thematic analysis method	• Contextual factors • Communication • Telemonitoring as support for managing the condition • The ‘fit' of telemonitoring with personal lifestyles and professional practice
Huygens et al. (44)	Netherlands	To investigate expectations and needs of people with a chronic condition regarding self-management and eHealth for self-management purposes, their willingness to use eHealth, and possible differences between patient groups regarding these topics	Patients with diabetes, COPD or cardiovascular condition	*N =* 30 (diabetes = 6, COPD = 9, cardiovascular condition = 7) Age range: 50–83 Mean age: 68	Content analysis method/Focus groups	• The impact of the chronic disease on patients' daily life • Their opinions and needs regarding self-management • Their expectations and needs regarding, and willingness to use, eHealth for self-management purposes
Gorst et al. (19)	UK	To explore the beliefs and perceptions of patients with chronic obstructive pulmonary disease currently using home telehealth and who are not enrolled in a trial	Patients with chronic obstructive pulmonary disease	*N =* 8 M = 3 F = 5 Age range: 58–84 Mean age: 64	Interpretative phenomenological analysis/Semi-structured interviews	• Perceiving benefits of “being watched over” as providing peace of mind • Learning about the health condition and the impacts on self-management behavior • Active engagement in health service provision and better access to healthcare • Valuing the importance of in-person care
Jiang et al. (32)	China	To explore the perceptions and experiences of older patients and healthcare providers in the application of telehealth and online health information to chronic disease management of chronic obstructive pulmonary disease	Patients with chronic obstructive pulmonary disease and healthcare providers	*N =* 52 (Patients = 29 Mean age: 70.6, HCPs = 23)	Inductive thematic analysis/Semi-structured interviews	• Faced with a vast • amount of online health information • Essential competencies and personality traits ensuring older patients' participation and sustained use • User experience with the use of technology • Being in a complex social context
Ladin et al. (35)	USA	To identify patients, care partners, and nephrologists' perceptions of the patient-centeredness, benefits, drawbacks of telehealth compared to in-person visits	Clinicians, patients with advanced kidney disease, and care partners	*N =* 60 (clinicians = 19, patients = 30, care partners = 11)	Thematic analysis/Semi-structured interviews/Phone interviews	• Variable Quality of Care • Patient Engagement With Clinicians • Loss of Interpersonal Connection and Mistrust • Difficulty of Breaking Bad News
Lyngå et al. (40)	Sweden	To explore and describe patients' perceptions of transmission of body weight (BW) and TM, regularly accomplished from patients' homes to an HF clinic	Patients with heart failure (HF)	*N =* 20 M = 15 F = 5	Phenomenological research design/Semi-structured interviews/Phenomenographic approach (Larsson and Holmstrom)	• The habitual patient • The concerned patient • The technical patient • The secure patient • The self-caring patient
Hanley et al. (21)	UK	To explore the experience of those taking part in the pilot trial and the views of a wider group of stroke survivors who may not have expressed interest in the trial	Patients with stroke or transient ischaemic attack (TIA) and carers	*N =* 46 (patients = 39, carers = 7)	Mixed methods feasibility study/Qualitative evaluation/Thematic analysis/Semi-structured interviews/Focus groups	• Usual care • Trying the new technology • Benefits and burdens of telemonitoring
Alkawaldeh et al. (34)	USA	To assess individuals' experiences and perceptions of using a tablet-based application for 30 days as a component of routine diabetes self-management care in older adults with type 2 diabetes mellitus (T2DM) in the context of daily living	Patients with T2DM who have been used a tablet-based application for 30 days	*N =* 12 F = 7 M = 5 Mean age = 68.7	Descriptive phenomenology/Semi-structured interviews/Thematic analysis (Braun & Clarke)	• Self-dependence • Awareness • Positive impact on attitude and behavior • Support • Balance
Ekstedt et al. (38)	Sweden	To explore patients' and healthcare professionals' experiences of safety and sense of security when using telemonitoring of chronic conditions at home	Patients with chronic conditions and healthcare professionals	*N =* 29 M = 11 F = 18 (patients = 20 Mean age: 72.5, HCPs = 9 Mean age: 52)	Inductive content analysis/Semi-structured interviews	• Increased availability enabled through telemonitoring • Having someone keep track of symptoms • The meeting with technology changed work and daily routines • Empowering patients' ability in self-management
Rahimpour et al. (45)	Australia	To identify any major factors that could affect patients' perceptions of a Home Telecare Management System (HTMS) and use the findings to contribute to the development of a theoretical framework for patient acceptance of HTMS	Patients with congestive heart failure (CHF), chronic obstructive pulmonary disease (COPD), or both	*N =* 77 Age range: 50–90 Mean age: 71	Focus group	• Intention to use the HTMS • The impact of the HTMS on patients' health management • Concerns associated with using the HTMS • The impact of the HTMS on healthcare services
Huniche et al. (46)	Denmark	To explore how chronic obstructive pulmonary disease (COPD) patients make use of readings during 16 weeks of self-monitoring in the Telekat project	Patients with chronic obstructive pulmonary disease	*N =* 22 M = 8 F = 14	German and Danish critical psychology/Semi-structured interview/Thematic analysis	• How readings are used by patients • Emotional responses to readings • How readings produce a sense of security
Poitras et al. (41)	Canada	To explore the perspective of patients with chronic diseases on teleconsultation in primary care	Patients with chronic diseases	*N =* 39 M = 16 F = 23 Mean age: 60.5	Collaborative longitudinal qualitative descriptive study/Semi-structured interview/Deductive thematic analysis	• Access to primary care clinics services during a pandemic • Advantages and disadvantages of teleconsultation compared with face-to-face encounters • Interprofessional collaboration • Healthcare professionals' competencies specific to teleconsultation • The patient-centered approach to care • Avenues for improving measures of patients' perceptions of their care experience • Patients' needs and preferences during a teleconsultation
Portz et al. (36)	USA	To use the Technology Acceptance Model (TAM) as a framework for qualitatively describing the UI and UX, intent to use, and use behaviors among older patients with MCC	Older adults with multiple Chronic Conditions	*N =* 24 M = 7 F = 17 Mean age: 78.41	Theoretically driven approach based on the TAM/Focus group/Semi-structured interviews	• Use behavior • Perceived ease of use • Computer anxiety • Computer self-efficacy • Perceived usefulness • Intent to Use
Poppe et al. (47)	Belgium	To explore how users with T2D experience ‘My Plan 2.0′, a theory-based eHealth intervention targeting physical activity and sedentary behavior	Patients with type 2 diabetes(T2D) who had completed an online self-regulation-based intervention(‘My Plan 2.0′)	*N =* 21 M = 13 F = 8 Age range: 57–81 Mean age: 65.86	Open-ended questions/Directed content analysis	• Usefulness of the website • Design of the website • Knowledge
Riley et al. (22)	UK	To explore the extent to which telemonitoring in patients with heart failure empowers them to self-care	Patients with heart failure	*N =* 15 M = 11 F = 4 Mean age: 44–86 Mean age: 74	Constant comparison (Strauss & Corbin)/In-depth interview	• The experience of symptoms • Use of the technology • Heart failure self-care maintenance activities • Heart failure self-management
Scheibe et al. (48)	Germany	To achieve intersectoral networking of treating physicians in practices, nurses, therapists, social services, and patients with multimorbidity and their caregivers based on the iterative development of a technology information and communication platform	Patients with multimorbidity and mild cognitive impairment (MCI)	*N =* 12 M = 4 F = 8 Mean age: 78.7	Structured content analysis/Open-ended questions	• Assessment of usability by patients with MCI • Additional benefits, negative effects, and changes in everyday life of patients with MCI • Ability of patients with MCI to use the telemonitoring app independently • Influence of previous experience With Smartphones, Tablets, or PCs on perceived ease of use of the telemonitoring app
Bond et al. (15)	UK	To evaluate a local telehealth program introduced by the Dorset Clinical Commissioning Group for patients with COPD or CHF	Patients with chronic obstructive pulmonary disease (COPD) or congestive heart failure (CHF) and healthcare professionals	*N =* 31 M = 16 F = 15	Focus group/Telephone interviews/Theoretical analysis	• Patients' experience and perception of the telehealth program • Healthcare professionals' experience and perception of the telehealth program
Seto et al. (42)	Canada	To assess the attitudes of heart failure patients and their health care providers from a heart function clinic in a large urban teaching hospital toward the use of mobile phone-based remote monitoring	Patients with heart failure clinicians	*N =* 36 (patients = 20, clinicia*N =* 16)	Mixed method/Semi-structured interviews/Conventional content analysis approach	• Willingness to use mobile phone-based remote monitoring • Ability to use mobile phone-based remote monitoring • Perceived benefits by patients and clinicians • Perceived barriers by patients and clinicians
Vatnøy et al. (49)	Norway	To explore how home-living patients diagnosed with chronic obstructive pulmonary disease (COPD) experienced follow-up using telemedicine, and the extent to which the implemented technology was able to support and improve the patients' coping resources and independence	Patients with COPD	*N =* 10 M = 7 F = 3	Semi-structured interviews/Qualitative content analysis (Graneheim and Lundman)	• The telemedicine solution is experienced as comprehensible and manageable and provides meaning in daily life • The telemedicine intervention contributes to stress reduction caused by illness burden and facilitates living as normally as possible
Hägglund et al. (39)	Sweden	To deductively test if the situation-specific theory of heart failure self-care could be applied in the context of persons with heart failure using a mHealth system with a tablet computer connected to a weighing scale to support their self-care	Patients with heart failure	*N =* 17 M = 11 F = 6 Mean age = 75	Semi-structured interviews/Deductive content analysis (Elo & Kyngas)	• Maintenance • Symptom perception • Management
Sultan et al. (43)	Canada	To identify specific challenges of patients with multiple chronic conditions and to use the findings to form design principles for a telemonitoring system tailored for these patients	Patients with multiple chronic conditions and clinicians	*N =* 25 (patients = 15, clinicians = 10)	Conventional content analysis/Semi-structured interview/Health belief model (HBM)	• Each patient has unique needs • Support for healthy lifestyle habits is required • Routines for self-management activities need to be established • Consolidating health information is burdensome • Patients need clinically accurate and actionable feedback • Communication and clinical integration must be efficient
Ure et al. (23)	UK	To explore the perceptions of patients and professionals about the pilot implementation of the COPD telemonitoring service	Patients with COPD, clinicians and managers	*N =* 45 (patients = 20, professionals = 25)	Mixed method/Face-to-face semi-structured interviews/Focus groups/Ethnographic observations	• Reducing delays in accessing care for exacerbations • Recognizing exacerbations or over-treatment? • Empowering self-care or increasing dependence? • Usability and reliability of technology • Use of healthcare resources
Williams et al. (24)	UK	To explore patients' expectations and experiences of using a mobile telehealth-based (mHealth) application and to determine how such a system may impact on their perceived well-being and ability to manage their COPD	Patients with chronic obstructive pulmonary disease (COPD)	*N =* 19 M = 11 F = 8 Mean age = 67 Age range: 50–80	Grounded theory approach/Constant comparative analysis	• Patients' transition from being uncertain about their ability to use technology to being confident to use it • The way in which the mHealth intervention addressed patient concerns about fragmented care by offering a sense of continuity of care and, thereby, reassurance • Increased patient awareness of the variability of symptoms; and • The way the mHealth intervention appeared to support patients' self-management behavior
Zulman et al. (37)	USA	To understand self-management and health care navigation challenges that patients face due to MCCs and to identify opportunities to support these patients through new and enhanced eHealth technology	Patients with ≥3 chronic conditions and experience using technology for health-related purposes	*N =* 53 M = 39 F = 14 Mean age: 59	Content analysis method/Focus group	• Patients with MCCs manage • a high volume of information, visits, and self-care tasks • They need to coordinate, synthesize, and reconcile health information from multiple providers and about different conditions • Their unique position at the hub of multiple health issues requires self-advocacy and expertise
Li et al. (27)	China	To explore the reasons why patients with hypertension refuse to use mobile medical platform, to provide reference for improving the platform content and implementing effective management for patients in the later stage	Patients with hypertension	*N =* 13 M = 7 F = 6 Age range: 31–58	Semi-structured interview/Colaizzi's seven-step analysis	• Resistance to new technology • Conscious of risk • Poor management effect, cumbersome operation when using • Feedback is not timely • Lack of new content • Frequent forgetting • Self-perception of good condition • Lack of social support
Lan et al. (25)	China	To explore the real feelings of patients with low willingness to accept heart failure mobile medical application (App) and the reasons for giving it up, to provide a basis for further improvement of mobile medical App for heart failure and improve intervention strategies	Patients with heart failure	*N =* 11 M = 7 F = 4	Semi-structured interview/Colaizzi's seven-step analysis	• Social demographic characteristics • Individual innovation characteristics • Perceived ease of use is not strong • Perceived usefulness is limited • There is a negative psychological experience affecting the use of intention
Tang et al. (26)	China	To explore the application experience of mobile medical platform for hypertension patients, to provide a reference for the continuous improvement and development of information platforms in the future	Patients with hypertension	*N =* 13 M = 7 F = 6 Mean age = 50.2 Age range: 22–71	Semi-structured interview/Colaizzi's seven-step analysis	• Disease management and health behavior change • Perceived differences in continued use • Identification and approval • Difficulties and concerns • Opinions and recommendations
Tang et al. (28)	China	To explore the needs of health education based on mobile health in community-dwelling older patients with Chronic Obstructive Pulmonary Disease (COPD)	Patients with COPD	*N =* 12 M = 10 F = 2	Semi-structured interview/Colaizzi's seven-step analysis	• The use needs of health education based on mobile health • The content needs of health education based on mobile health • The functional needs of health education based on mobile health • The design needs of health education based on mobile health
Zhang et al. (29)	China	To understand the demands of patients with COPD for tele-pulmonary rehabilitation nursing, and to provide the theoretical basis for the construction of a tele-pulmonary rehabilitation nursing model in China.	Patients with COPD	*N =* 14	Phenomenological approach/Semi-structured interview/Colaizzi's seven-step analysis	• Self-care demands • Social support demands • Information system demands
Zhou et al. (30)	China	To understand the use experience of smart pill box for older patients with chronic disease in community, and to provide new ideas for further enhancing user stickiness and optimizing smart pill box	Patients with chronic conditions	*N =* 1 M = 9 F = 7	Semi-structured interview/Colaizzi's seven-step analysis	• “Promoting health” • “Affirming the function of Smart Pill Box” • “Low willingness to embrace” • “Technology anxiety”

HCPs, healthcare professionals; F, Female; M, Male; COPD, chronic obstructive pulmonary disease; HF, heart failure; CHF, congestive heart failure; MCC, multiple chronic conditions; TIA, transient ischemic attack; T2DM, type 2 diabetes mellitus.

According to the JBI quality assessment scale, 27 studies achieved grade B, and 8 studies achieved grade A (Table 2). Most studies did not describe the investigator's impact on the study. Some studies do not describe the researcher's cultural and theoretical orientation.

**Table 2:** JBI quality assessment results of the included primary studies.

**References**	**Is there congruity between the stated philosophical perspective and the research methodology?**	**Is there congruity between the research methodology and the research question or objectives?**	**Is there congruity between the research methodology and the methods used to collect data?**	**Is there congruity between the research methodology and the representation and analysis of data?**	**Is there congruity between the research methodology and the interpretation of results?**	**Is there a statement locating the research culturally or theoretically?**	**Is the influence of the researcher on the research, and vice-versa, addressed?**	**Are participants, and their voices, adequately represented?**	**Is the research ethical according to current criteria or, for recent studies, and is there evidence of ethical approval by an appropriate body?**	**Do the conclusions drawn in the research report flow from the analysis, or interpretation, of the data?**	**Grade**
Lu et al. (33)	Y	Y	Y	Y	Y	Y	Y	Y	Y	Y	A
Chang et al. (31)	Y	Y	Y	Y	Y	Y	Y	Y	Y	Y	A
Fairbrother et al. (17)	Y	Y	Y	Y	Y	U	Y	Y	Y	Y	B
Fairbrother et al. (16)	Y	Y	Y	Y	Y	U	Y	Y	Y	Y	B
Fairbrother et al. (18)	Y	Y	Y	Y	Y	U	Y	Y	Y	Y	B
Hanley et al. (21)	Y	Y	Y	Y	Y	U	U	Y	Y	Y	B
Huygens et al. (44)	Y	Y	Y	Y	Y	U	Y	Y	Y	Y	B
Gorst et al. (19)	Y	Y	Y	Y	Y	Y	Y	Y	Y	Y	A
Jiang et al. (32)	Y	Y	Y	Y	Y	Y	U	Y	Y	Y	B
Ladin et al. (35)	Y	Y	Y	Y	Y	U	Y	Y	Y	Y	B
Hanley et al. (20)	Y	Y	Y	Y	Y	Y	U	Y	Y	Y	B
Alkawaldeh et al. (34)	Y	Y	Y	Y	Y	U	Y	Y	Y	Y	B
Ekstedt et al. (38)	Y	Y	Y	Y	Y	Y	Y	Y	Y	Y	A
Rahimpour et al. (45)	Y	Y	Y	Y	Y	Y	Y	Y	Y	Y	A
Huniche et al. (46)	Y	Y	Y	Y	Y	U	Y	Y	Y	Y	B
Poitras et al. (41)	Y	Y	Y	Y	Y	U	Y	Y	Y	Y	B
Portz et al. (36)	Y	Y	Y	Y	Y	U	Y	Y	Y	Y	B
Poppe et al. (47)	Y	Y	Y	Y	Y	U	U	Y	Y	Y	B
Riley et al. (22)	Y	Y	Y	Y	Y	Y	U	Y	Y	Y	B
Scheibe et al. (48)	Y	Y	Y	Y	Y	U	Y	Y	Y	Y	B
Bond and Worswick (15)	Y	Y	Y	Y	Y	U	U	Y	Y	Y	B
Seto et al. (42)	Y	Y	Y	Y	Y	U	U	Y	Y	Y	B
Vatnøy et al. (49)	Y	Y	Y	Y	Y	Y	Y	Y	Y	Y	A
Hägglund et al. (39)	Y	Y	Y	Y	Y	U	U	Y	Y	Y	B
Sultan et al. (43)	Y	Y	Y	Y	Y	Y	Y	Y	Y	Y	A
Ure et al. (23)	Y	Y	Y	Y	Y	U	U	Y	Y	Y	B
Williams et al. (24)	Y	Y	Y	Y	Y	U	U	Y	Y	Y	B
Zulman et al. (37)	Y	Y	Y	Y	Y	U	Y	Y	Y	Y	B
Li et al. (27)	Y	Y	Y	Y	Y	U	U	Y	Y	Y	B
Lan et al. (25)	Y	Y	Y	Y	Y	U	Y	Y	Y	Y	B
Tang et al. (26)	Y	Y	Y	Y	Y	Y	Y	Y	Y	Y	A
Tang et al. (27)	Y	Y	Y	Y	Y	U	Y	Y	Y	Y	B
Zhang et al. (29)	Y	Y	Y	Y	Y	U	U	Y	Y	Y	B
Zhou et al. (30)	Y	Y	Y	Y	Y	U	Y	Y	Y	Y	B

N, No; U, Unclear; Y, Yes.

### 3.3 Data analysis and meta-synthesis

Eight sub-themes were extracted from the 35 included studies. Through meta-synthesis, ten sub-themes were compared and analyzed, and four themes were identified. The needs and improvement suggestions for telemedicine by patients with chronic diseases can be divided into four themes: (1) reminders and supervisors, (2) access to knowledge, (3) transition in medical treatment mode, and (4) emotional support platform. Data are presented as a synthesized finding with supporting themes and component subthemes. Themes with key exemplary quotations are presented below and in Table 3.

**Table 3:** Key supporting quotes per theme.

**Themes**	**Supporting quotes**
**1. Reminder and supervisor**	
Sense of security	“I just find it reassuring that I can check manually what my oxygen levels are, because I'm aware of the fact that I get anxious about things and everything goes to pot, so it's reassuring. I think that's the biggest positive…I know my children like the fact that I've got it. They are very much aware of the fact that I do not look after myself and so it reduces the worry for them.” (16) “The disadvantage is that I'm feeling more like a patient [because of frequently monitoring]: man suffers most from the suffering he fears.” (41) “The platform is real-name system, my ID number and home address are inside, I am a little concerned about the security of information.” (23) “Unless it is regulated by the government … only this can prevent fraud and information leakage. Then people can trust the internet.” (29)
Improving disease management capabilities	“It's made it easier for me to know what's what. If you don't have the machine then you don't have any facts to talk about. So I suppose it's made me realize I have to do certain things in a certain way and when I've felt like perhaps experimenting I know I mustn't, with the medication for instance.” (19) “It makes me feel more confident. It's good up to a point you think you're doing really good. It forces me to do everything, and it feels good. It gives me more energy because I want to keep track of it.” (31)
**2. Access to knowledge**	
Improving health literacy	“I wanted to know what to pay attention to in general (for COPD patients). At the hospital, the doctors were too busy to talk about these stuff. I had to look it up on the internet myself. It was said that tai chi could be good for COPD, so I have tried it.” (29) “I value the characteristics of your Chinese medicine so I am willing to join you in this management, I believe in some of the healthcare methods of Chinese medicine, and the doctors in the platform can be found in the hospital, I am very relieved, compared with other software on the market, I trust you more.” (23)
Patients' concerns about knowledge	“Online rehab exercises for COPD… I would consider whether it would be good for my health. If so, I would like to follow it… If online health information said I needed to take medication, I would be more cautious… looking it up on the internet to check or asking my doctor. What if I took the wrong medication and messed up my body.” (29) “Some articles in the mobile phone feel too professional, such as some guidelines and pharmacological knowledge, I am really not easy to understand. Some of the text is too much to read.” (22)
**3. Transition in medical treatment mode**	
Convenience	“It suits my needs [teleconsultation] because I don't have to spend money on gas.” (38) “On the phone, [clinicians are] more tender than they are in person.” Patient 36 thought that “maybe the doctor is more committed to providing his time because of [telehealth]…we're able to have a greater dialogue…than at the office.” (32) “How can doctors conduct a follow-up consultation online?…without listening by a stethoscope and tapping by fingers. Just saying a few words… without a physical examination, there must be something wrong with the diagnosis.” (29)
Multidisciplinary coordination	“…so with the system like this they will be able to send the data to each other (such as general practitioner and specialist) quickly.” (42) “Somebody phoned me to say, ‘We're not happy with your blood pressure reading, you've got to go and see your doctor…make an appointment to see your doctor…' He just said, ‘Oh it's fine…your blood pressure is fine now'.” (17)
**4. Emotional support platform**	
Peer support	“You have that WeChat group is quite good, and the successful experience of patients will help us a lot.” (26)
Family support	“What was relevant to telehealth is that the patient might have a relative at home with them [making] the logistics [are] a lot easier.” (32)

#### 3.3.1 Reminder and supervisor

##### 3.3.1.1 Sense of security

Most patients consider peace of mind to be one of the biggest benefits of using telemedicine, as they can see some physiological indicators of their body at any time and understand the progress of their condition, which reduces patients' worry and uncertainty. At the same time, telemedicine brings more security to patients because they know that healthcare professionals will check their data regularly, and the doctor will take the initiative to contact them if there is a problem in time (15, 17–20, 22–24, 26, 33, 34, 36, 38, 42, 44–46). Patients also said that telemedicine can reduce the concerns of patients' families.

“*I just find it reassuring that I can check manually what my oxygen levels are, because I'm aware of the fact that I get anxious about things and everything goes to pot, so it's reassuring. I think that's the biggest positive…I know my children like the fact that I've got it. They are very much aware of the fact that I do not look after myself and so it reduces the worry for them.” (19)*

However, some patients who use telemonitoring at home may have anxiety about the accuracy of the data and how to handle abnormal data, which is one of the reasons that affect patients' willingness to use telemedicine. One *cardiovascular* patient said that he was frequently reminded of having a chronic condition.

“*The disadvantage is that I'm feeling more like a patient [because of frequently monitoring]: man suffers most from the suffering he fears.” (44)*

People with chronic conditions worry about their personal and health information being improperly accessed or misused by non-medical personnel. Frequent news about data breaches makes their doubts about the security of telemedicine (26, 27, 32, 44, 45).

“*The platform is real-name system, my ID number and home address are inside, I am a little concerned about the security of information.” (26)*

Patients and HCPs emphasized that standard regulation of information by government departments was the only way to relieve people's prejudices about telemedicine.

“*Unless it is regulated by the government … only this can prevent fraud and information leakage. Then people can trust the internet.” (32)*

##### 3.3.1.2 Improving disease management capabilities

Self-management of chronic diseases is a complex and ongoing process. Telemedicine helps to improve patients' self-management abilities and encourages self-management behaviors. By monitoring the dynamic changes in data, patients can understand their physical condition, which will motivate them to take the initiative in disease management (15, 17, 19–24, 26, 30, 31, 33, 34, 40, 43, 45, 46). The patient said that telemedicine taught her to rely on professional data for self-management.

“*It's made it easier for me to know what's what. If you don't have the machine then you don't have any facts to talk about. So I suppose it's made me realize I have to do certain things in a certain way and when I've felt like perhaps experimenting I know I mustn't, with the medication for instance.” (22)*

In addition, both good measurement data and praise from healthcare professionals will increase patient confidence and positive perceptions of telemedicine, thus encouraging patients to maintain good self-management and strengthening the user stickiness of telemedicine. A 62-year-old patient with type 2 diabetes said:

“*It makes me feel more confident. It's good up to a point you think you're doing really good. It forces me to do everything, and it feels good. It gives me more energy because I want to keep track of it.” (34)*

#### 3.3.2 Access to knowledge

##### 3.3.2.1 Improving health literacy

Formal education and training are an important part of patient self-management. Patients often do not know how to manage their disease because of a lack of knowledge about treatment and care. Most patients said their doctors had not told them or told them about their illness when they were in the hospital, but they forgot about it when they were discharged. As a new way to acquire knowledge, patients can query disease-related knowledge and learn proper disease management methods through telemedicine, thus improving their health literacy (17, 19, 24, 28, 30–32, 36, 37, 44).

“*I wanted to know what to pay attention to in general (for COPD patients). At the hospital, the doctors were too busy to talk about these stuff. I had to look it up on the internet myself. It was said that tai chi could be good for COPD, so I have tried it.” (32)*

Specialized knowledge boards can strengthen patients' willingness to use telemedicine. Tang et al. (26) mentioned TCM-related (Traditional Chinese Medicine-related) functions that were popular with patients.

“*I value the characteristics of your Chinese medicine so I am willing to join you in this management, I believe in some of the healthcare methods of Chinese medicine, and the doctors in the platform can be found in the hospital, I am very relieved, compared with other software on the market, I trust you more.”*

##### 3.3.2.2 Patients' concerns about knowledge

Faced with a large amount and variety of health knowledge, patients feel that they are unable to judge the reliability and applicability of information. In addition, patients said that much popular science knowledge was too specialized, and they could not understand it. All these have greatly hindered patients' use of telemedicine (18, 25, 28, 30, 32, 39, 43, 47).

“*Online rehab exercises for COPD… I would consider whether it would be good for my health. If so, I would like to follow it… If online health information said I needed to take medication, I would be more cautious… looking it up on the internet to check or asking my doctor. What if I took the wrong medication and messed up my body” (32)*

“*Some articles in the mobile phone feel too professional, such as some guidelines and pharmacological knowledge, I am really not easy to understand. Some of the text is too much to read.” (25)*

#### 3.3.3 Transition in medical treatment mode

##### 3.3.3.1 Convenience

In addition to reducing patient travel time and transportation costs to the hospital, telemedicine also allows doctors to telecommute and give timely feedback to patients. Telemedicine realizes the remote communication between patients and medical staff, provides health services such as consultation and diagnosis for patients, and is conducive to the establishment of a healthy and harmonious doctor-patient relationship (15–23, 26, 28, 30, 32, 33, 35–39, 41, 42, 45, 46, 49). Patients said doctors were gentler when they used telemedicine to talk to them.

“*It suits my needs [teleconsultation] because I don't have to spend money on gas” (41)*

“*On the phone, [clinicians are] more tender than they are in person.” Patient 36 thought that “maybe the doctor is more committed to providing his time because of [telehealth]…we're able to have a greater dialogue…than at the office.” (35)*

However, telemedicine is only a supplement and continuation of the traditional medical treatment model. Given the lack of face-to-face communication and examination in remote assessment, some patients and doctors believe this may generate some negative conditions, including missed diagnoses, misdiagnoses, and a lack of intimacy (24, 32, 35, 41).

“*How can doctors conduct a follow-up consultation online?…without listening by a stethoscope and tapping by fingers. Just saying a few words… without a physical examination, there must be something wrong with the diagnosis.” (32)*

##### 3.3.3.2 Multidisciplinary coordination

Telemedicine can facilitate the exchange of patient health-related information among healthcare professionals. By uniting communities and hospitals, patients can be provided with comprehensive continuity of care, which facilitates chronic disease management and follow-up.

“*…so with the system like this they will be able to send the data to each other (such as general practitioner and specialist) quickly.” (45)*

However, a lot of patients thought they may be under the care of multiple doctors, who would work independently of each other. Because they operate as separate entities, they often do not have complete information about a patient's condition or treatment history, leading to fragmented and poorly coordinated care for patients (16, 18, 20, 36, 43).

“*Somebody phoned me to say, ‘We're not happy with your blood pressure reading, you've got to go and see your doctor…make an appointment to see your doctor…' He just said, ‘Oh it's fine…your blood pressure is fine now'.” (20)*

#### 3.3.4 Emotional support platform

##### 3.3.4.1 Peer support

“Prolonged illness makes the patient a doctor.” Patients with chronic disease usually develop a relatively comprehensive and in-depth understanding of their condition after a long period of disease management and treatment, and they may become advocates for their health, sharing experiences with others. Telemedicine provides a platform for patients to communicate or organize online support groups, allowing patients to communicate and connect with other patients with similar conditions. They can share experiences, support, enhance mental health, and improve motivation for each other (26, 27, 29, 30, 37).

“*You have that WeChat group is quite good, and the successful experience of patients will help us a lot.” (29)*

##### 3.3.4.2 Family support

Family support plays a vital role in the self-management of patients with chronic diseases. In the face of complicated treatment and lifestyle adjustment, patients need understanding and help from family members both psychologically and practically. Telemedicine allows family members to monitor the patient's health data in real time, acknowledge the patient's condition and treatment progress, and participate in medical decision-making. Meanwhile, by encouraging and assisting patients to install and use telemedicine applications, family members can reduce patients' fear and resistance to new technologies, and significantly improve patients' acceptance and frequency of use of telemedicine (19, 26, 27, 30, 32, 35, 39, 46–48). HCPs emphasize the importance of family support:

“*What was relevant to telehealth is that the patient might have a relative at home with them [making] the logistics [are] a lot easier.” (35)*

## 4 Discussion

This study explores, compares and synthesizes the experience of using telemedicine for chronic patients. While previous studies have mostly focused on patient experiences and preferences, they have failed to systematically articulate suggestions for improvements in telemedicine. Therefore, from the perspective of functional modules, this study reveals four comprehensive themes, which summarize the application functions and improvement suggestions of telemedicine in chronic disease management.

Our study identified the alerting and monitoring functions of telemedicine as the first comprehensive topic. Consistent with Creber's study, patients with chronic diseases improved their knowledge of their disease through telemedicine, which improved their self-care ability and sense of security (10). Telemedicine also improves long-term adherence to self-management in patients with chronic diseases and is a promising way to support patients and clinicians in treating their disease. An RCT study showed that an innovative telemonitoring enhanced care program for Chronic heart failure (ITEC-CHF) improved patient adherence to weight monitoring (50). However, some chronic patients will worry about telemedicine's accuracy and individual information safety. Providing successful cases and establishing the legal framework to protect patients' interests and rights, then enhances the authority and trust of the telemedicine platform. People with chronic diseases often have a dismissive attitude toward their illness, believing that they can control it on their own. Research shows that old age, low income, low education, and other factors affect patients' willingness to use telemedicine (51). Telemedicine should formulate personalized programs for them according to the individual's age, education level, and other aspects. Consistent with other reviews on telemedicine (7, 10), patients question the effectiveness of telemedicine. Existing research is confused about the effectiveness of telemedicine. A systematic review and meta-analysis (52) reported that telemedicine had the potential to improve clinical outcomes for people with diabetes, but the overall quality of its evidence was low to very low. Further prospective studies are needed to gather clinical evidence supporting the effectiveness of telemedicine.

Telemedicine is often used by patients to gain health knowledge. Telemedicine improves patient self-management by providing daily education and health information. The self-management needs of people with chronic illnesses are varied. Telemedicine should focus on the individual needs and preferences of patients, avoid a lot of homogenized information, carry out diversified health education in form and content, recommend personalized knowledge to patients (31), and meet their health needs, which helps to enhance their confidence and sense of control over chronic diseases (53), and thus promote the sustainable development of telemedicine. In addition, patients may question the reliability of health knowledge and the security of personal information. For the sustainable and healthy development of telemedicine, the platform should strengthen information review to ensure the reliability of knowledge. Policymakers should further improve the network information security guarantee mechanism, strengthen the construction of laws and regulations on telemedicine, and ensure the reliability of health information (54). Healthcare professionals should use plain language to provide health education to patients and take the initiative to answer questions related to privacy protection, so as to provide patients with reliable sources of health information, improve their health literacy, and enhance patients' sense of trust (55).

Telemedicine has changed the traditional medical model. Compared with face-to-face methods, telemedicine reduces the time and transportation costs for patients and facilitates remote access to healthcare guidance for patients with chronic diseases, especially those with limited mobility and financial difficulties. Telemedicine can also improve the efficiency of healthcare professionals, promote the sharing of patient health information, provide comprehensive continuity of care for patients, and facilitate the management and follow-up of chronic diseases. This is consistent with Yi's research (56) that telemedicine has a positive effect on chronic disease management. However, telemedicine cannot replace face-to-face consultations. The study found that although telemedicine is widely used in most medical specialties, only a few specialties have strong evidence that the diagnostic and management decisions provided by telemedicine are comparable to face-to-face care (57). Telemedicine is simply an adjunct to medical practice, supporting the work of healthcare professionals when needed. However, telemedicine also poses challenges for healthcare professionals. The use of telemedicine by healthcare professionals often results in members working independently, lack of communication, inability to ensure consistency of treatment plans (41, 43) and poor communication with patients. Therefore, telemedicine should be integrated into the entire healthcare system as a tool to enhance the continuity of the relationship between hospitals, patients, and communities. Develop skills training to improve the remote consultation capacity of healthcare professionals and combine community health resources to ensure continuity of care and improve the working mode of telemedicine. Strengthening the integration of telemedicine with multidisciplinary collaboration allows healthcare professionals from different disciplines to share patient information through electronic health records, video conferencing, and online platforms to improve diagnostic accuracy and quality of care (58).

Vorderstrasse et al. (59) found that telemedicine provides people with type 2 diabetes with interactions with peers, family members, and healthcare professionals that can help them address the challenges and barriers that arise when implementing self-management behaviors. When incorporating telemedicine into the self-management pathway for patients with chronic diseases, healthcare professionals should provide a multifunctional information exchange platform to involve patients and families in care and enhance patient confidence in self-management and treatment compliance.

## 5 Implications for clinical practice and future studies

The qualitative evidence synthesis of telemedicine in chronic disease self-management explored the application of telemedicine in the field of chronic disease self-management from the functional module, providing a new perspective for telemedicine research. The results show that telemedicine is an adjunct tool to support clinical practice and is conducive to improving patient satisfaction and quality of care. However, the effectiveness of telemedicine needs further research to explore. This study provides recommendations for stakeholders, policymakers, and healthcare professionals to address current issues in telemedicine. Regarding information leakage and online medical insurance, policymakers should introduce effective personal information protection systems and fair medical insurance policies to promote the sustainable development of telemedicine. The hospital realizes data sharing and business collaboration with telemedicine services, and actively provides continuous services such as convenient and efficient online services, follow-up management and remote guidance for patients. Joint community hospitals promote hierarchical diagnosis and treatment and carry out the monitoring and management of the characteristic data of patients with chronic diseases, to reduce medical costs and improve the level of primary medical services. Telemedicine skills training is carried out to improve the communication, interface operation and data processing skills of healthcare professionals, which is conducive to improving the quality of medical care. Future research efforts should prioritize assessing the needs of people with chronic diseases, considering their countries, digital literacy, and healthcare environments. Such an assessment would help improve the feasibility and sustainability of telemedicine. It is worth emphasizing that 77% of chronic disease deaths occur in low—and middle-income countries, but much of the research on telemedicine in chronic disease management originated in high-income countries (1). In low and middle-income countries, there is relatively little research. Low—and middle-income countries should further strengthen telemedicine research to harness its potential more effectively, improve health services and reduce the impact of chronic diseases (60).

## 6 Limitations

The limitation of this review is that it is limited to papers published in both Chinese and English. Another limitation is that the study is from different countries, and there are differences in patients' cultural backgrounds, and telemedicine devices, which may make it more difficult for researchers to integrate information and affect the quality of the findings. In addition, the operation and cost of telemedicine are not discussed in this review, because we believe that simplifying operation is inconsistent with the concept of fully understanding patients' conditions and providing personalized services for them. Inter-device charging standards and the variation of healthcare systems between countries are too large, stakeholders should solve relevant questions according to national conditions. Technical limitations are a barrier to the adoption of telemedicine in rural areas. However, most of the literature included in this study is from developed countries with fewer technical restrictions, so we do not discuss them in depth.

## 7 Conclusion

This study systematically reviewed the content of telemedicine promoting chronic disease self-management and deeply discussed the positive role and improved suggestions of telemedicine in chronic disease self-management. Through the Technology Acceptance Model (TAM) (61), patients are provided with operational training and successful cases, and a clear legal regime is established to enhance the authority and trust of the telemedicine platform. In addition, improves the medical service system, establishes a multidisciplinary coordination mechanism, introduces corresponding telemedicine information protection policies, and encourages information sharing among hospitals at all levels, to improve the willingness of patients and hospitals to use telemedicine.
