# Supplementary material for: Exploring the impact of telemedicine in chronic patients from diverse socioeconomic contexts: systematic review of qualitative studies
Source: Front Public Health. 2025 Jan 29;12:1510735. doi: 10.3389/fpubh.2024.1510735 (PMC11813940; doi:10.3389/fpubh.2024.1510735)
Supplement: Supplementary file 2 [file Data_Sheet_2.docx]

**Supplementary File 2: Search strategy**

| **Examples of search strategy** | | |
| --- | --- | --- |
| **PubMed** | | |
| #1 | "chronic diseases"[Title/Abstract] | 52204 |
| #2 | "Telemedicine"[MeSH Terms] OR "electronic health"[Title/Abstract] OR "Mobile Health"[Title/Abstract] OR "electronic therapy"[Title/Abstract] OR "mHealth"[Title/Abstract] OR "Telehealth"[Title/Abstract] OR "eHealth"[Title/Abstract] OR "Telecare"[Title/Abstract] OR "Telenursing"[Title/Abstract] OR "etherapy"[Title/Abstract] | 110638 |
| #3 | "Qualitative Research"[MeSH Terms] OR "qualitative study"[Title/Abstract] OR "Qualitative Research"[Title/Abstract] OR "qualitative method"[Title/Abstract] OR "interview*"[Title/Abstract] OR "experience*"[Title/Abstract] OR "feeling*"[Title/Abstract] OR "perception*"[Title/Abstract] OR "attitude*"[Title/Abstract] | 2301502 |
| #4 | #1 AND #2 AND #3 | 410 |
| **Embase** | | |
| #1 | 'chronic disease':ab,ti | 65317 |
| #2 | 'telemedicine':ab,ti | 29317 |
| #3 | 'electronic health':ab,ti | 52523 |
| #4 | 'Mobile Health':ab,ti | 7965 |
| #5 | 'electronic therapy':ab,ti | 14 |
| #6 | 'mHealth':ab,ti | 7421 |
| #7 | 'Telehealth':ab,ti | 18207 |
| #8 | 'eHealth':ab,ti | 9414 |
| #9 | 'Telecare':ab,ti | 1057 |
| #10 | 'Telenursing':ab,ti | 299 |
| #11 | 'etherapy':ab,ti | 108 |
| #12 | #2 OR #3 OR #4 OR #5 OR #6 OR #7 OR #8 OR #9 OR #10 OR #11 | 114952 |
| #13 | 'qualitative study':ab,ti | 80825 |
| #14 | 'qualitative research':ab,ti | 25074 |
| #15 | 'qualitative method':ab,ti | 2766 |
| #16 | 'interview*':ab,ti | 624984 |
| #17 | 'experience*':ab,ti | 2107037 |
| #18 | 'feeling*':ab,ti | 127784 |
| #19 | 'perception*':ab,ti | 435333 |
| #20 | 'attitude*':ab,ti | 257631 |
| #21 | #13 OR #14 OR #15 OR #16 OR #17 OR #18 OR #19 OR #20 | 3085209 |
| #22 | #1 AND #12 AND #21 | 475 |
| **Web of science** | | |
| #1 | TS=("chronic diseases") | 110443 |
| #2 | TS=("Telemedicine" OR "Mobile Health" OR "electronic health" OR "electronic therapy" OR "electronic therapy" OR "electronic therapy" OR "mHealth" OR "Telehealth" OR "eHealth" OR "Telecare" OR "Telenursing" OR "etherapy") | 238172 |
| #3 | TS=("qualitative study" OR "qualitative research" OR "qualitative method" OR "interview*" OR "experience*" OR "feeling*" OR "perception*" OR "attitude*") | 9231484 |
| #4 | #1 AND #2 AND #3 | 6298 |
| **Cochrane** | | |
| #1 | MeSH descriptor: [chronic diseases] explode all trees | 44052 |
| #2 | (chronic diseases):ti,ab,kw | 30057 |
| #3 | #1 OR #2 | 65950 |
| #4 | MeSH descriptor: [Telemedicine] explode all trees | 5100 |
| #5 | (Mobile Health OR electronic health OR electronic therapy OR electronic therapy OR electronic therapy OR mHealth OR Telehealth OR eHealth OR Telecare OR Telenursing OR etherapy):ti,ab,kw | 42011 |
| #6 | #5 OR #6 | 44553 |
| #7 | MeSH descriptor: [Qualitative Research] explode all trees | 2434 |
| #8 | (qualitative study OR qualitative research OR qualitative method OR interview* OR experience* OR feeling* OR perception* OR attitude*):ti,ab,kw | 259872 |
| #9 | #7 OR #8 | 259873 |
| #10 | #3 AND #6 AND #9 | 766 |
| **Scopus** | | |
| #1 | TITLE-ABS-KEY ("chronic diseases") | 419412 |
| #2 | TITLE-ABS-KEY ("Telemedicine" OR "Mobile Health" OR "electronic health" OR "electronic therapy" OR "electronic therapy" OR "electronic therapy" OR "mHealth" OR "Telehealth" OR "eHealth" OR "Telecare" OR "Telenursing" OR "etherapy") | 199969 |
| #3 | TITLE-ABS-KEY ("qualitative study" OR "qualitative research" OR "qualitative method" OR "interview*" OR "experience*" OR "feeling*" OR "perception*" OR "attitude*") | 6247530 |
| #4 | #1 AND #2 AND #3 | 2072 |
| **CNKI** | | |
| #1 | TKA=(慢性病) | 1183816 |
| #2 | TKA=(移动医疗 + 医疗保健 + 远程医疗 + 智慧医疗) | 51025 |
| #3 | TKA=(质性研究 + 定性研究) | 62978 |
| #4 | #1 AND #2 AND #3 | 19 |
| **VIP** | | |
| #1 | M=(慢性病) | 97318 |
| #2 | M=(移动医疗 OR 医疗保健 OR 远程医疗 OR 智慧医疗) | 995149 |
| #3 | M=(质性研究 OR 定性研究) | 2145590 |
| #4 | #1 AND #2 AND #3 | 712 |
| **WanFang** | | |
| #1 | 题名或关键词:(慢性病) | 36671 |
| #2 | 题名或关键词:(移动医疗 OR 医疗保健 OR 远程医疗 OR 智慧医疗) | 26633 |
| #3 | 题名或关键词:(质性研究 OR 定性研究) | 412323 |
| #4 | #1 AND #2 AND #3 | 3 |
